# Hepatitis E virus (HEV) in Scotland: evidence of recent increase in viral circulation in humans

**DOI:** 10.2807/1560-7917.ES.2018.23.12.17-00174

**Published:** 2018-03-22

**Authors:** Katrina Thom, Pamela Gilhooly, Karen McGowan, Kristen Malloy, Lisa M Jarvis, Claire Crossan, Linda Scobie, Oliver Blatchford, Alison Smith-Palmer, Mhairi C Donnelly, Janice S Davidson, Ingolfur Johannessen, Kenneth J Simpson, Harry R Dalton, Juraj Petrik

**Affiliations:** 1 Scottish National Blood Transfusion Service, Edinburgh, United Kingdom; 2 Department of Biological and Biomedical Sciences, School of Health and Life Sciences, Glasgow Caledonian University, Glasgow, United Kingdom; 3 Department of Public Health, Glasgow University, Glasgow, United Kingdom; 4 Health Protection Scotland, National Services Scotland, Glasgow, United Kingdom; 5 Department of Hepatology, Division of Health Sciences, Edinburgh Medical School, Edinburgh, United Kingdom; 6 Scottish Liver Transplantation Unit, Royal Infirmary, Edinburgh, United Kingdom; 7 Department of Laboratory Medicine, NHS Lothian, Edinburgh, United Kingdom; 8 Royal Cornwall Hospital and European Centre for Environment and Human Health, University of Exeter Medical School, Truro, United Kingdom

**Keywords:** Hepatitis E virus (HEV), epidemiology, seroprevalence, incidence, zoonosis, blood donors

## Abstract

Previous studies showed low levels of circulating hepatitis E virus (HEV) in Scotland. We aimed to reassess current Scottish HEV epidemiology. **Methods:** Blood donor samples from five Scottish blood centres, the minipools for routine HEV screening and liver transplant recipients were tested for HEV antibodies and RNA to determine seroprevalence and viraemia. Blood donor data were compared with results from previous studies covering 2004–08. Notified laboratory-confirmed hepatitis E cases (2009-16) were extracted from national surveillance data. Viraemic samples from blood donors (2016) and chronic hepatitis E transplant patients (2014–16) were sequenced. **Results:** Anti-HEV IgG seroprevalence varied geographically and was highest in Edinburgh where it increased from 4.5% in 2004–08) to 9.3% in 2014–15 (p = 0.001). It was most marked in donors < 35 years. HEV RNA was found in 1:2,481 donors, compared with 1:14,520 in 2011. Notified laboratory-confirmed cases increased by a factor of 15 between 2011 and 2016, from 13 to 206. In 2011–13, 1 of 329 transplant recipients tested positive for acute HEV, compared with six cases of chronic infection during 2014–16. Of 10 sequenced viraemic donors eight and all six patients were infected with genotype 3 clade 1 virus, common in European pigs. **Conclusions:** The seroprevalence, number of viraemic donors and numbers of notified laboratory-confirmed cases of HEV in Scotland have all recently increased. The causes of this change are unknown, but need further investigation. Clinicians in Scotland, particularly those caring for immunocompromised patients, should have a low threshold for testing for HEV.

## Introduction

Hepatitis E virus (HEV) is now increasingly recognised in industrialised countries, where it causes acute infection, and chronic infection in immunosuppressed individuals, including transplant recipients [1,2]. In Europe, locally acquired hepatitis E is predominantly a porcine zoonosis associated with HEV genotypes (gt) 3 and 4. Around 70% of individuals exposed to HEV gt3 are asymptomatic [3]. In many industrialised countries infection with HEV is more common than previously thought. For instance, based on blood donor data from South East England, there are an estimated 100,000 human infections each year in England [4]. Because HEV infection is common and commonly asymptomatic, HEV has found its way into the blood supply, with frequency of viraemic donations between 1:600 to 1:14520 [5-7] and recently some countries (France, Germany, Ireland, the Netherlands) have started or are considering the implementation of HEV RNA donation screening [8,9]. This includes the United Kingdom (UK), which introduced screening of donations destined for ‘high-risk’ recipients (solid organ and haematological stem cell transplant recipients, neonates) [10] in February 2016 and universal screening in March 2017.

Anti-HEV IgG seroprevalence and incidence of hepatitis E varies both between and within countries, and over time. For example, in south-west (SW) France, one study has found a seroprevalence of 52% and incidence of hepatitis E of 3% [11]. Although HEV appears to be hyperendemic in SW France, seroprevalence varies considerably between French departments (range: 8–86%) [12]. The reason for this observation is unknown, but could be related to geographical differences in dietary habits. In contrast, seroprevalence in Scotland, first assessed for the period 2004–08, was found to be 4.7% [5]. In recent years the number of cases of hepatitis E documented in many European countries, including Scotland, has increased, mostly due to improved testing and case ascertainment. However, there is evidence that points to a recent genuine increase in incidence in some European countries [6,12-16].

Compared with many European countries, previously reported Scottish seroprevalence data and rates of viraemia in blood donors were low [5]. In an earlier study, donors were sampled during 2004–08 and were mainly from the Edinburgh area. However, figures for the rest of Scotland and recent changes in seroprevalence and numbers of viraemic blood donors are unknown. The aims of this study were to determine the recent seroprevalence in differing geographical areas of Scotland and if there has been a change in recent years and to cross reference this with the current numbers of viraemic donors and laboratory-confirmed cases, including those in the Scottish liver transplant population.

## Methods

### Study population and laboratory tests performed

An overview of the different data sets and periods covered that were included in this study together with laboratory tests performed is given in Table 1. Further details are described in specific sections hereafter.

**Table 1:** Sample and data sets used in this study

**Sample origin**					
	**Sample type**	**Collection period**	**Tested for**	**Number tested**	**Reference**
Anonymous archive; Five Scottish blood donation centres covering the whole country	Plasma	August 2014–September 2015	HEV IgG	1,714	This study
Routine HEV RNA screening	Plasma minipools of 24	February 2016–May 2017	HEV RNA	94,302	This study
Liver transplant recipients	Serum	2011–13	HEV RNA, IgG and IgM	329	This study
Liver transplant recipients	Serum	2014–16	HEV RNA, IgG, IgM	NA	This study
**Dataset origin**					
	**Data included**	**Collection period**	**Tested for**	**Number** **tested**	**Reference**
Health Protection Scotland	Notified laboratory-confirmed HEV cases	2009–16	Hepatitis E	NA	[17,18]
Anonymous archive of Scottish blood donations, samples from Edinburgh area	Results from previous study	2004–08	HEV IgG, IgM	1,559	[5]
Routine testing samples	Results from previous study	2011	HEV RNA	43,560	[5]
HEV test performed, RI Edinburgh	Testing data	2010–11; 2016	HEV RNA, IgG, IgM	243 (2010–11) 1,153 (2016)	This study

HEV: hepatitis E virus; NA: not available; RI: Royal Infirmary.

#### Case definitions

A case of acute hepatitis E was defined as: anti-HEV IgM and IgG positive and/or HEV RNA detection by RT-PCR. A case of chronic hepatitis E was defined as: HEV RNA detected by PCR in serum and/or stool persisting for a minimum of 3 months. A case of previous HEV infection was defined as: anti-HEV IgG positive, and anti-HEV IgM negative.

#### Blood donor serology samples

Following donor consent, plasma samples were obtained from the Scottish National Blood Transfusion Service (SNBTS) research anonymous archive, a project allowing epidemiological surveillance/look-back in Scottish blood donors. Samples were collected from August 2014 to September 2015 at the five existing blood collection centres in Scotland. Data provided with the samples were age, sex, first part of post code and whether the donor was a repeat or first-time donor. The serology data were analysed in two ways. First, we compared the anti-HEV seroprevalence in Edinburgh, with our previous data (2004–08) from the same area [5] using the same serological assay to determine changes over time. Second, we compared the anti-HEV seroprevalence by geographical region across Scotland.

Sera were tested for anti-HEV IgG using the Wantai kit (Beijing Wantai Biological Pharmacy Enterprise Co, Beijing, China), following the manufacturer’s instructions. Borderline results were treated as negative.

#### Blood donor RNA screening samples

As part of the national screening programme, a proportion of Scottish blood donors were tested for HEV RNA from February 2016 and all donations from March 2017. In general, collections were geographically aligned with population demographics, with more donations collected in the central belt (i.e. the densely populated area between Glasgow and Edinburgh). There was no significant blood group bias (data not shown). We report the first 15 months of data generated by the screening programme in Scotland, i.e. February 2016 to May 2017.

Samples from blood donors in the screening programme were tested in pools of 24 donations using the Cobas HEV test on the Cobas 6800 system (Roche, Pleasanton, United States (US)). Confirmatory testing involved testing for HEV RNA and anti-HEV IgG and IgM. Nucleic acid was extracted on an EasyMAG semi-automated nucleic extraction system (Biomerieux, Marcy l’Etoile, France) followed by the individual donation amplification and detection using the ampliCube HEV 2.0 kit (Mikrogen Diagnostik, Neuried, Germany). Samples containing HEV RNA were sequenced as described below.HEV NAT screening of blood donations is performed in pools of 24 donations using the cobas HEV test on the cobas 6800 system (Roche). Confirmatory testing involves testing for HEV RNA and HEV IgG and IgM antibodies. Nucleic acid is extracted on an easyMAG semi-automated nucleic extraction system (Biomerieux) followed by amplification and detection using the ampliCube HEV 2.0 kit (Mikrogen Diagnostik).

#### Notified laboratory-confirmed cases

HEV is a notifiable infection in Scotland and Health Protection Scotland publishes the number of laboratory-confirmed cases each year as part of routine national surveillance [17,18]. Data from 2009 onwards were included in the study.

#### Hepatitis E cases in Scottish liver transplant recipients

Liver transplant recipients attending the Scottish Liver Transplant Unit for routine post-transplant follow-up during 2011–13 were tested for HEV in a cross-sectional cohort study. Following informed consent, a blood sample was taken and stored at −70^0^C until tested. From 2014 onwards, liver transplant recipients were tested for HEV when clinically indicated. The indications for HEV testing in such patients included persistently raised transaminase levels, suspected drug-induced liver injury and hepatitis on liver biopsy which was otherwise unexplained.

#### RT-PCR, sequencing and phylogenetic analysis of positive blood donor and liver transplant recipient samples

Reverse transcription (RT)-PCR was performed using the Access RT-PCR System and nested PCR using GoTaq (Promega Corporation, Madison, WI, US). Primers 3156 and 3157 [19] for the primary reaction and 3158 [19] and TAQR [5] for the secondary reaction and the amplification conditions were as described [19]. Amplified DNA was gel-purified using QIAEX II gel extraction kit (Qiagen, Hilden, Germany) and sequenced using Mix2Seq kit (Eurofins, Wolverhampton, UK). The sequence alignment and phylogenetic analysis were carried out using Mega7 package [20,21]. 358 base pair (bp) sequences corresponding to the nested PCR product, and a set shortened to 219 bp, allowing inclusion of the shorter three sequences from our first study [5] and from donor 4. Ten viraemic donor samples and six patient samples were sequenced as at October 2016.

In addition, four overlapping regions were amplified from a plasma sample of an acutely infected transplant recipient and an implicated blood donor sample using LA Taq reagents (Clontech, Saint-Germain-en-Laye, UK) and primers as described [22]. Multiple overlapping reads were assembled using DNA Dragon (Sequentix, Klein Raden, Germany).

### Ethics

Ethical permission was obtained from the Scottish Medical Research and Ethics Committee 10/MREC/00/74, West of Scotland Research Ethics Committee (15/WS/0081) and the SNBTS sample governance committee.

### Statistics

Chi-squared test and chi-squared test for trend or Fisher's exact test were performed, as indicated, to compare proportions of seropositive samples in different groups using GraphPad Prism version 7.01 for Windows (GraphPad Software, La Jolla California US).

## Results

### Blood donor seroprevalence

One thousand seven hundred and fourteen blood donor samples (835 female, 879 male donors) were included from Aberdeen, Dundee, Edinburgh, Glasgow and Inverness, (Table 2). Of these, 104 were HEV IgG seropositive (50 female, 54 male donors), giving an overall seroprevalence of 6.1% (95% confidence interval (CI): 5.0–7.3). There was no difference between the seropositivity of male and female donors (6.1%; 95% CI: 4.6–7.9 and 6.0%; 95% CI: 4.5–7.8, respectively).

**Table 2:** Blood donor demographics and hepatitis E virus seroprevalence by sex, five regional blood collection centres, Scotland, August 2014–September 2015 (n=1,714)

Donor centre	Sex	Donors (n)	Median age (years)	Age range (years)	HEV IgG positive (n)	HEV IgG seroprevalence (%)	HEV IgG seroprevalence 95% CI (%)
**Aberdeen**	**F**	126	40.5	19–66	6	4.8	2.2–10.0
	**M**	169	43	17–70	5	3.0	1.3–6.7
	**Total**	295	42	17–70	11	3.7	2.1–6.6
**Dundee**	**F**	164	46.5	19–73	12	7.3	4.2–12.4
	**M**	152	50	18–68	12	7.9	4.6–13.3
	**Total**	316	49	18–73	24	7.6	5.2–11.1
**Edinburgh**	**F**	207	47	19–70	16	7.7	4.8–12.2
	**M**	221	47	17–74	20	9.1	5.9–13.6
	**Total**	428	47	17–74	36	8.4	6.1–11.4
**Glasgow**	**F**	173	45	17–71	8	4.6	2.4–8.9
	**M**	174	49	17–74	7	4.0	2.0–8.1
	**Total**	347	47	17–74	15	4.3	2.6–7.0
**Inverness**	**F**	165	47	18–73	8	4·9	2.5–9.3
	**M**	163	50.5	17–72	10	6.1	3.4–10.9
	**Total**	328	49	17–73	18	5.5	3.5–8.5
**All sites combined**	**F**	835	45	17–73	50	6.0	4.6–7.8
	**M**	879	48	17–74	54	6.1	4.7–7.9
	**Total**	1,714	47	17–74	104	6.1	5.0–7.3

CI: confidence interval; HEV: hepatitis E virus.

Seroprevalence differences Edinburgh vs Glasgow (p=0.03), Edinburgh vs Aberdeen (p= 0.02) and Dundee vs Aberdeen (p= 0.04) were statistically significant.

**Figure 1:**
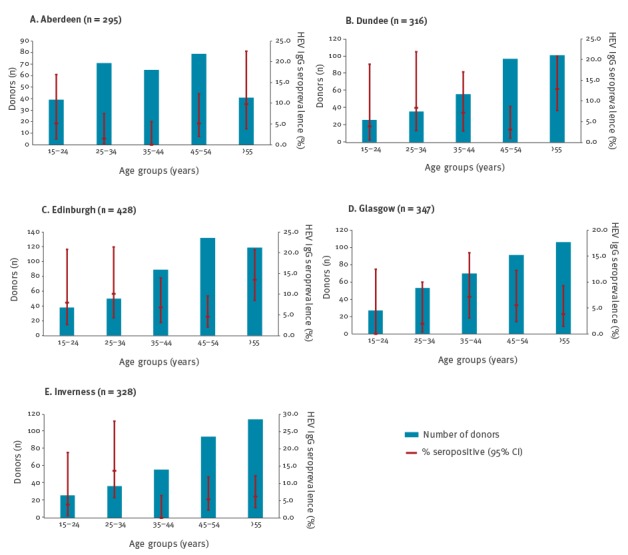
Anti-hepatitis E virus IgG seroprevalence in differing areas by age, Scotland, August 2014 to September 2015 (n=1,714)

There was a significant variation of seroprevalence between some of the donor centres (Table 2). It was higher in Edinburgh (8.4%) and Dundee (7.6%), compared with Aberdeen (3.7%), Glasgow (4.3%) and Inverness (5.5%). Seroprevalence by age (Figure 1) demonstrated somewhat different patterns from the usual gradual increase from the youngest to oldest blood donors. Seroprevalence among donors in the 25–34 year age group was higher than in next two age groups (35–44 and 45–54-year-olds) in Edinburgh, Inverness and Dundee, and the seroprevalence in over the 55 year-olds age group was not the highest in Glasgow and Inverness.

CI: confidence interval.

The age-specific seroprevalence is shown; 1.17% of the total cohort were first time donors.

**Figure 2:**
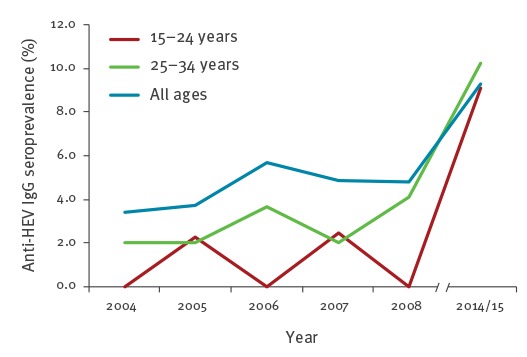
Anti-HEV seroprevalence by age in Edinburgh, Scotland 2004–2015

During the years 2004–08, the seroprevalence in Edinburgh residents was 4.5% (95% CI: 3.5–5.8) increasing to 9.3% (95% CI: 4.3–20.2) in the period 2014–15 (p = 0.001, Figure 2).

Seroprevalence of donors donating at the Edinburgh blood donation centre who live in Edinburgh, as defined by their residential post code starting with the letters EH. Note that 2004–08 data are derived from the dataset in the study of donors donating at the Edinburgh centre [5], patients with post codes other than EH were excluded.

Increases in seroprevalence were mainly seen in younger donors, with increases of 9.5 times in 15–24-year-olds and 3.7 times for 25–34 year age group (Figure 2). The combined seroprevalence in donors < 35 years of age increased fivefold between 2004 and 2008 (2.0%; 95% CI: 0.1–8.1) and 2014–15 (9.8%; 95% CI: 4.3–20.2; p = 0.0005). The seroprevalence in the 25–34 year-olds in 2014–15 crossed the overall seroprevalence (all ages) for the same time period, indicating increased exposure to HEV in this age group compared with the rest of the cohort. The proportion of seropositive donors who were aged > 35 years decreased from 85% in 2004–08 to 77% in 2014–15.

### Hepatitis E viraemia in screened donors

Nucleic acid testing of 94,302 blood donations (51,388 from selective testing February 2016 to February 2017 and 42,914 from universal testing March to May 2017) detected HEV RNA in 38 donors (1:2,481), of which 23 (1:2,234) were detected during selective screening and 15 (1:2,860) during universal screening. Of these donors, 29 had negative anti-HEV serology, eight were positive for both anti-HEV IgG and IgM and one had HEV IgM antibodies only. The mean age of the viraemic donors was 44 years and 6 months (median 47 years; range 21–69) and three donors were less than 30 years old. Twenty-one of the viraemic donors resided in an area with a Glasgow post code, the remaining 17 donated at Edinburgh (n=8), Dundee (n=6) and Aberdeen (n=3) centres. No positive donations collected at Inverness centre were detected up until May 2017. More HEV infections were detected in male donors (n = 27) than female donors (n = 11). Sequence analysis was possible for 10 of these donations (see below).

### Notified laboratory-confirmed cases of hepatitis E in Scotland

**Figure 3:**
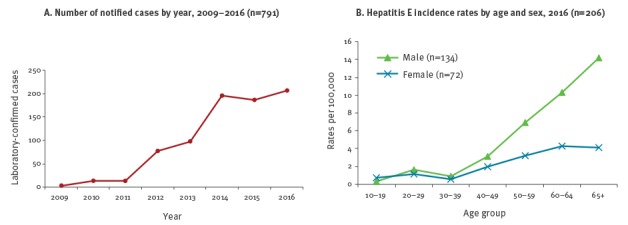
Notified laboratory-confirmed cases of hepatitis E, Scotland

There has been a rapid increase in the annual incidence of laboratory-confirmed cases of hepatitis E from 13 cases in 2011 to 206 cases in 2016 (Figure 3A), giving a current overall incidence rate of hepatitis E in Scotland of 3.8 cases per 100,000 population, with a predominance of infection in older males (Figure 3B). In 2015, there were 124 (67.6%) male cases and of these 61 (45.4%) were aged 65 years and older [17,18].

Data published by Health Protection Scotland [17,18].

### Number of hepatitis E virus tests performed

Data on the number of HEV tests performed were only available from Edinburgh. In 2010–11, 243 patients were tested for HEV. Of these, 10 (4.3%) fulfilled our case definition. In contrast, in 2016, 1,153 patients were tested for HEV. Of these, 74 (6.4%) fulfilled our case definition.

### Hepatitis E cases in Scottish liver transplant recipients

In 2011–13, a total of 329 Scottish liver transplant recipients were tested for HEV by serology and PCR. Patients ranged in age from 18 to 85 years (mean: 56.4; SD 13.6) with a male to female ratio of 1:1.07. The time post-transplant was up to 20 years (mean: 5.0; SD: 5.3). No case of chronic HEV infection was documented.

In contrast, between 2014 and 2016, six patients were identified with chronic HEV infection following liver transplantation in Scotland. HEV infection was diagnosed between 5 to 105 months following liver transplantation. In four of these patients, a trial of immunosuppression reduction was undertaken, in an attempt to prompt clearance of HEV, which was successful in one case. Five patients, including three still on reduced immunosuppression, required antiviral therapy with ribavirin, and successful viral clearance was achieved in four cases.

One case of acute hepatitis E was found in 2012, in a recipient 68 days post-liver transplant. He had no symptoms of hepatitis and samples taken earlier confirmed that the recipient was HEV RNA-negative before transplantation. Evidence of HEV infection in the donated liver was absent. The recipient had received 32 blood components (37 donor exposures) during transplant surgery and examination of archived samples from these blood components identified a donor of fresh frozen plasma with active HEV infection (HEV IgM and HEV RNA positive, HEV IgG negative). Phylogenetic analysis of the sequence HEV species from the HEV RNA-positive blood donation and the liver transplant patient revealed gt3, clade 1/group 2 virus (GenBank accession number KT159771). Highly conserved match between the virus from the donor and patient (99.6–99.8% homology), indicated a transfusion transmitted infection.

### Sequencing data

**Figure 4:**
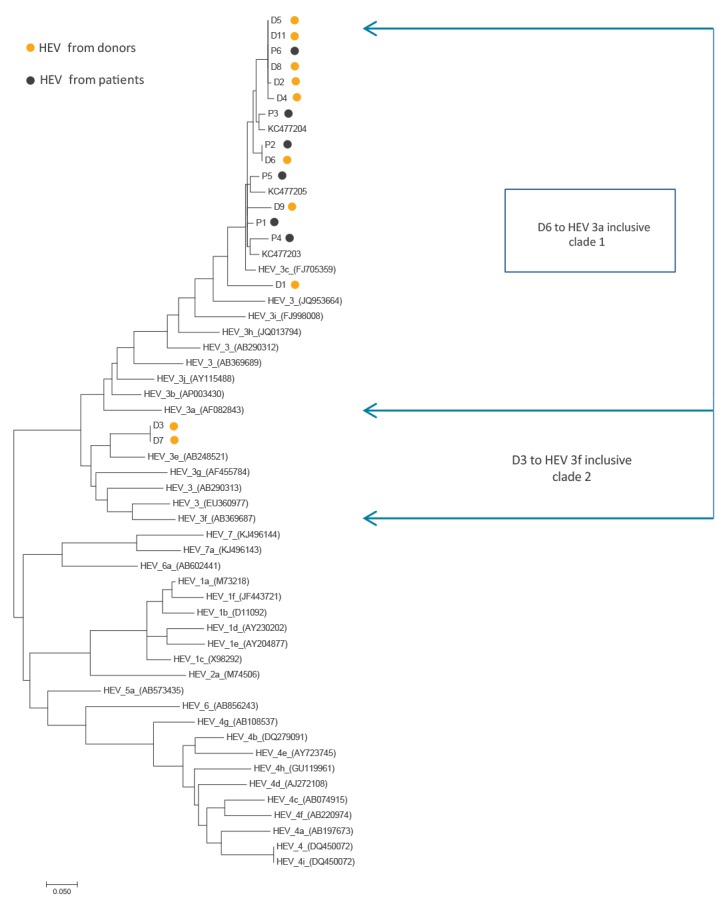
Phylogenetic tree of hepatitis E virus sequences from Scottish donors and patients

Eight of ten donor sequences and all six 2014 to 2016 liver transplant recipients’ sequences grouped with gt 3 clade 1/group 2. Sequencing data from the donors and patients are shown in Figure 4.

D: donor; Gt: genotype; HEV: hepatitis E virus; p: patient.

HEV from donors (D) in 2016 and liver transplant patients (P) infected in 2014–16. KC477203–205: sequences of positive samples from donor minipools collected in 2011 [5]. The evolutionary history was inferred by using the maximum likelihood method based on the Tamura-Nei model [20]. The tree with the highest log likelihood (-3861.1232) is shown. Initial tree(s) for the heuristic search were obtained automatically by applying neighbour-joining and BioNJ algorithms to a matrix of pairwise distances estimated using the maximum composite likelihood (MCL) approach, and then selecting the topology with superior log likelihood value. The tree is drawn to scale, with branch lengths measured in the number of substitutions per site. The analysis involved 55 nt sequences. Codon positions included were 1st + 2nd + 3rd + non-coding. All positions containing gaps and missing data were eliminated. There were a total of 219 positions in the final dataset. Evolutionary analyses were conducted in MEGA7 [21]. GenBank accession numbers for donor and chronic HEV patients’ sequences are KY197769–83. Phylogenetic trees based on longer, 324 and 358 bp sequences (excluding shorter KC477203–205 [5] and D4 sequences) gave the same pattern.

## Discussion

The data presented here suggest that there has been a significant and recent increase in circulating HEV in Scotland. Analysis of samples from blood donors showed that the number of viraemic donors rose from 1:14,520 to 1:2,481 from 2011 to 2016-17, accompanied by a rise in seroprevalence in Edinburgh, particularly in younger donors. Between 2011 and 2016, the number of notified laboratory-confirmed HEV cases increased by a factor of 15. Prior to 2014, no case of chronic infection was found in the Scottish liver transplant recipient population. In 2014 to 2016, however, six cases of chronic infection were documented. In Edinburgh, both the number of cases tested for HEV and the percentage of positive tests increased. This suggests that, in addition to increased clinician awareness and testing, there was a true increase in circulating HEV in humans, at least in Edinburgh.

Two of the more intriguing outcomes of this study relate to the unexpected increase in seroprevalence in younger donors in Edinburgh and viraemia rates in screened blood donors from the general Scottish blood donor cohort. Taken together, these data suggest that there may be more circulating HEV in the Scottish community than hitherto was the case. The reason for this change is uncertain, but increases in incidence/seroprevalence have recently also been seen in England [15], France [12] and most compellingly, the Netherlands where the seroprevalence in young adults has increased steeply and the number of viraemic donors moved up from 1:2,671 in 2011 [23] to 1:600 in 2014 [6].

In England and Wales, temporally-related to the increase in incidence, strains of HEV gt3 recovered from humans have also changed. Previously, HEV in human cases bore close sequence homology to UK pigs (gt3 e,f,g; clade 2/group 1, but more recently appears to be mostly similar to HEV gt3 found in pigs in the UK and continental Europe (gt3 a,b,c,h,i,j; clade 1/group 2) [15,24,25]. The sequencing data presented in the current paper shows that HEV isolated from Scottish donors and patients were largely gt3 clade 1/group 2. One possibility to account for these observations could be imported HEV-contaminated foodstuffs from abroad i.e. continental Europe. However, recent sequence data from pigs in Scotland are scarce and the source and route of infection in studied patients and blood donors in Scotland remain uncertain. A study in England and Wales, by Said et al. suggested that recent human infections with HEV gt3 clade 1/group 2 may largely be due to consumption of food products from a single national supermarket chain [26]. Some of the possible speculative reasons for the increased seroprevalence in younger Scottish donors include a preferred choice of certain foodstuffs in younger people and/or ways of preparation, perhaps favouring faster cooking, including food preparation during outdoor social activities.

The findings suggesting that considerable amounts of HEV circulate in the Scottish community, need to be considered in the light of previous studies. Previously, anti-HEV IgG seroprevalence in Scotland was very low by European standards (4.7%), viraemic donors were uncommon (1:14,520) and numbers of notified laboratory-confirmed cases were very low before 2011. Analyses from the west of Scotland (2008–09) showed that only two of 316 patients with unexplained hepatitis had hepatitis E (data not shown). Prior to 2014, no cases of chronic infection were documented in the Scottish liver transplant population, but in the last two years, six cases of chronic infection with HEV have been documented, only five of which have meanwhile achieved viral clearance. These data underscore the clinical relevance of hepatitis E in immunosuppressed patients.

As has been shown elsewhere, we also found that HEV seroprevalence varied geographically within Scotland. In France, the overall seroprevalence is higher than that seen in Scotland and is highest in south-western, southern and north-eastern France [12]. In Scotland, the seroprevalence is highest in Edinburgh (8.4% overall; SE Scotland) and lowest in Aberdeen (3.7% overall; NE Scotland). The reason for such differences is unknown. It seems unlikely to be due to local environmental contamination from HEV-containing porcine faeces, as the main pig-rearing area in Scotland is Aberdeenshire [27] and in France it is Brittany, both of which have relatively modest anti-HEV IgG seroprevalence compared with other areas in the respective countries. It seems more likely that differences in seroprevalence are related to regional differences in exposure to contaminated products in the food chain. In France, local culinary delicacies include figatellu and air-dried Toulousean pig liver sausage. Both of these have been reported to be linked to HEV infection via consumption, with HEV detected sometimes at high viral loads [11,28]. Consumption of these foodstuffs are highest in southern France, which may account (at least in part) for the very high seroprevalence in this region. Although HEV has been found in Scottish pigs [29] and shellfish [30], differences in local culinary culture in Scotland are perhaps less marked than in France, and much of the food supply chain (i.e. via supermarkets) is on a national scale and so the reason(s) for regional differences in seroprevalence remains unclear. It is of note that the total pig herd in Scotland decreased from 470,000 in 2005 to 318,000 in 2015 (a drop of 32%) [27]. Although seroprevalence varies by location in Scotland, viraemic donors were found from diverse locations from across the country, and the increased seroprevalence in young adults was common to most of the locations where donors were tested. Taken together, these data suggest that the increase in circulating HEV we have documented may be a national rather than regional phenomenon.

The current study has a number of limitations. There are few current sequencing data on HEV in Scottish pigs, so we cannot be certain that imported pork from abroad is the source of infection, as was hypothesised to be the case in England and Wales. Furthermore, we have no data on travel history in the patients and donors. We have only presented data on the change of seroprevalence in Edinburgh, and we cannot be certain that the finding of increased viral occurrence in humans in this locality is a national phenomenon across the rest of Scotland. Notified laboratory-confirmed cases are well known to underestimate the true number of infections with any microorganism. This is particularly so for infection with HEV which is frequently asymptomatic. Although a highly sensitive and specific serology assay was applied across all tested groups, results obtained by PCR were more heterogeneous. To determine viraemic donations, two methods in minipools of 24 were used: in-house method in a 2013 study [5], and a commercial Roche assay (see Methods), in this study. Since the commercial assay is more sensitive, we have checked the detection capability of the in-house method using the first 20 positive samples detected in the course of routine testing by the commercial assay. Seventeen of 20 positive results were confirmed by the in-house method, indicating that the reported sharp increase in number of viraemic donations was not simply due to the use of different methods.

Finally, our clinical data from the transplant population are rather heterogeneous and since 2014 have relied on clinician awareness, which almost certainly varies from clinician to clinician. Our findings should thus be approached with these limitations in mind.

## Conclusion

The current situation in Scotland is unique with high levels of circulating virus in a largely HEV-naïve population. The source(s) and route(s) of infection in Scotland are unknown and require further epidemiological investigation. Increased numbers of infection are already occurring, which are likely to be mostly asymptomatic, but not necessarily without clinical impact, as demonstrated by the data on six chronic infections in Scottish liver transplant recipients in the years 2014–2016. Clinicians in Scotland, particularly those caring for immunocompromised patients, should have a low threshold for testing patients for HEV.
